# Paediatric inflammatory multisystem syndrome temporarily associated with SARS‐CoV‐2: It is not over until it's over!

**DOI:** 10.1111/jpc.16097

**Published:** 2022-07-01

**Authors:** Rania Haydar, Andrew M Davis, Bennett Sheridan, Edward Buratto, Georgia Brown, Bryn Jones

**Affiliations:** ^1^ Department of Pediatric Intensive Care Unit Royal Children's Hospital Melbourne Victoria Australia; ^2^ University of Melbourne Melbourne Victoria Australia; ^3^ Department of Pediatric Intensive Care Unit Murdoch Children's Research Institute Melbourne Victoria Australia; ^4^ Present address: 56/166 Sydney Street, New Farm, 4005 Brisbane QLD Australia

**Key points**

1. Cardiac arrhythmia, including advanced atrioventricular block, is being increasingly recognised as a rare complication of paediatric inflammatory multisystem syndrome‐temporarily associated with SARS‐CoV‐2 (PIMS‐TS).^1^, ^2^, ^3^
2. Recent cases of arrhythmia highlight the importance of performing a baseline electrocardiogram in all patients presenting with a picture consistent with PIMS‐TS regardless of absent cardiac involvement from the outset. This will allow changes to be noted early, permitting rapid anticipation of complications such as arrhythmia and heart block.
3. The medical facility should have the capacity to perform emergent ventricular pacing in severe cases.
4. During the convalescent phase, monitoring of heart rate and oxygen saturation should continue, and symptoms such as syncope require prompt investigations.
5. The WHO clinical management COVID‐19 guideline 2021,^4^ recommends the use of corticosteroids in addition to supportive care (rather than either IVIG plus supportive care, or supportive care alone). We note that our patient did not receive ‘early’ steroids. It is worth noting that we do not know if ‘early’ steroid administration may reduce the risk of cardiac arrhythmia.

## Case Report

A 7‐year‐old girl of African descent, with no history of symptoms consistent with COVID‐19, presented to our quaternary paediatric centre with a maculopapular rash and painful red eyes, preceded by a 3‐day prodrome of fever (40.3°C), vomiting and diarrhoea. She was tachycardic with a heart rate of 160 beats per min and had anterior cervical lymphadenopathy. Initial investigations demonstrated a mild metabolic acidosis (pH 7.27), lactate of 2.7 mmol/L, raised inflammatory markers with an erythrocyte sedimentation rate (ESR) of 107 mm/h, c‐reactive protein (CRP) of 206 mg/L and procalcitonin (PCT) of 18.7 μg/L. There was a mild coagulopathy with an international normalised ratio (INR) of 1.4, activated partial thromboplastin (APTT) of 56 s, prothrombin time (PT) 17.4 s, fibrinogen of 5.6 g/L, a mild lymphopenia at 0.95 × 10^9^/L and thrombocytopenia with a platelet count of 133 × 10^9^/L. Liver and kidney function were unremarkable. Initial ferritin was raised at 462 μg/L but troponin I was normal at 0.03 μg/L. Electrocardiogram (ECG) showed sinus tachycardia, with a Pwave to Rwave (PR) interval of 154 ms. Given the nature of the hyper‐inflammatory presentation, differential diagnoses included paediatric inflammatory multisystem syndrome‐temporarily associated with SARS‐CoV‐2 (PIMS‐TS) or multisystem inflammatory syndrome in children (MIS‐C), Toxic shock syndrome, atypical Kawasaki syndrome and Macrophage activation syndrome. Initial management included supportive care, empiric antibiotics, aspirin (125 mg) and intravenous immunoglobulin (IVIG) 2 g/kg. COVID results included: SARS‐CoV‐2 PCR negative on multiple Nasopharyngeal/Oropharyngeal swabs, bronchoalveolar lavage and faecal specimens. SARS‐CoV‐2 serology:IgG positive by Immunofluorescence (titre 1:40) and Liaison IgG EIA. IgM and IgA negative by Immunofluorescence.

**Fig. 1:**
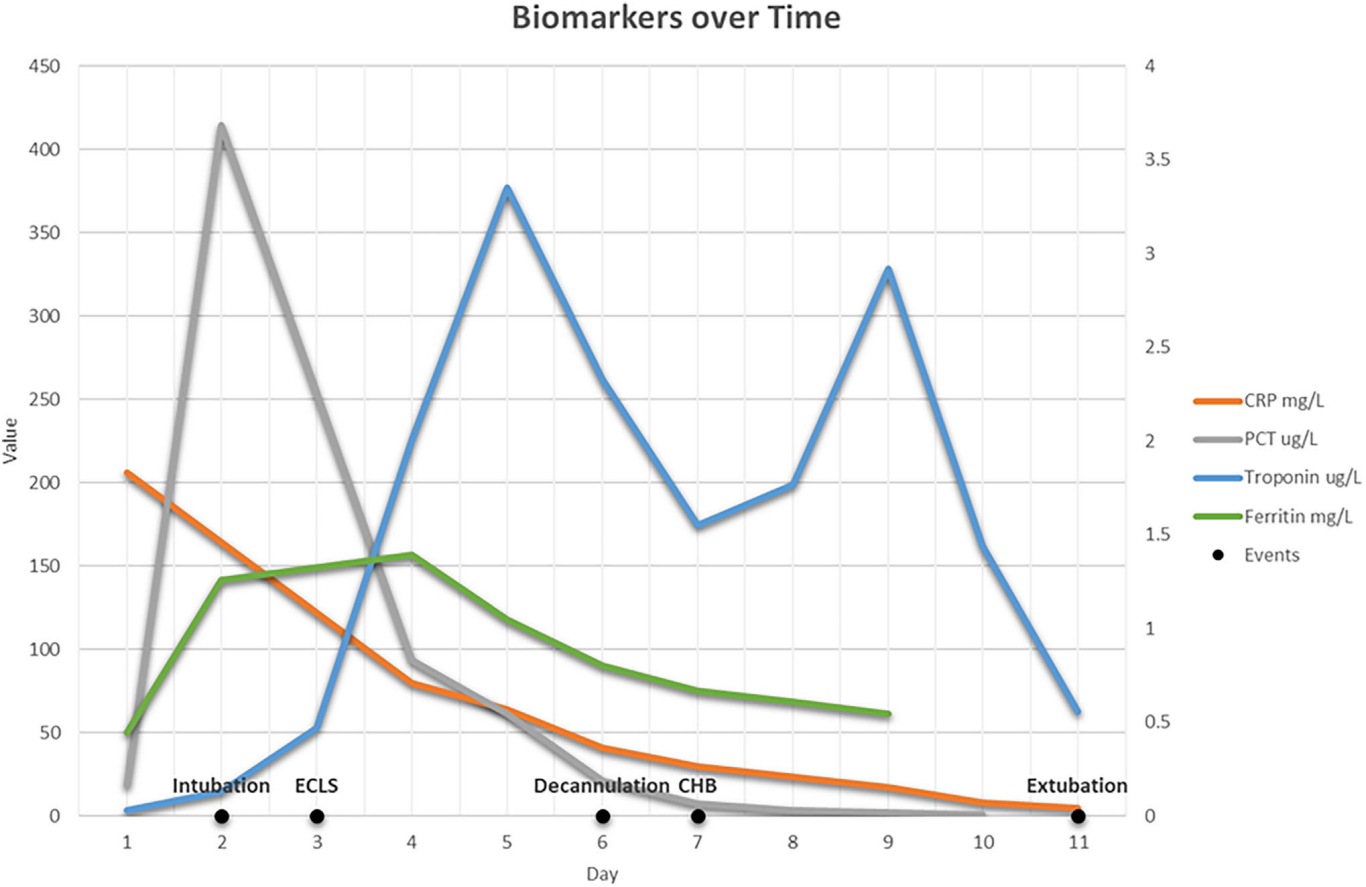
Graph of inflammatory markers over time.

Over the first 24 h, venous blood gases demonstrated a persistent metabolic acidosis, lactatemia and a marked rise in inflammatory markers with a mixed venous saturation (MVSO_2_) of 40% (Fig. 1). Twelve hours after admission, the patient was transferred to ICU for progressive haemodynamic compromise and commenced on an adrenaline infusion. Echocardiography demonstrated no pericardial effusion, mildly depressed left ventricular (LV) systolic function, no coronary ectasia or aneurysm formation. A second dose of IVIG 2 g/kg, pulse methylprednisolone 10 mg/kg and tocilizumab (12 mg/kg) were administered.

Within 30 h of hospital presentation, the patient was intubated, ventilated, and despite escalating inotropes and vasopressors demonstrated cold shock with a worsening metabolic acidosis, lactatemia and an arterial–venous difference (oxygen extraction ratio) of 52%. An echocardiogram confirmed severe LV dysfunction. Within 10 h of intubation, extracorporeal life support (ECLS) was semi‐urgently instituted by central cannulation (18Fr aortic cannula: dual‐stage right atrial venous cannula). High flow ECLS (200 mls/kg) was achieved, consistent with institutional practice for sepsis.^5^ Despite achieving high flow ECLS with inotropic support, the aortic valve remained closed with progressive LV dilation, necessitating left atrial (LA) vent insertion. An acute Troponin rise occurred around the time of vent insertion but resolved over the following days. By 72 h of admission, ferritin had peaked, but other inflammatory markers had begun to reduce (Fig. 1). After 105 h of ECLS, an epicardial echocardiogram performed at low ECLS flow again demonstrated normal coronary arteries with improved LV function. Levosimendan was administered in preparation for removal of LA vent and decannulation from ECLS, which occurred on day 6 of admission.^6^

**Fig. 2:**
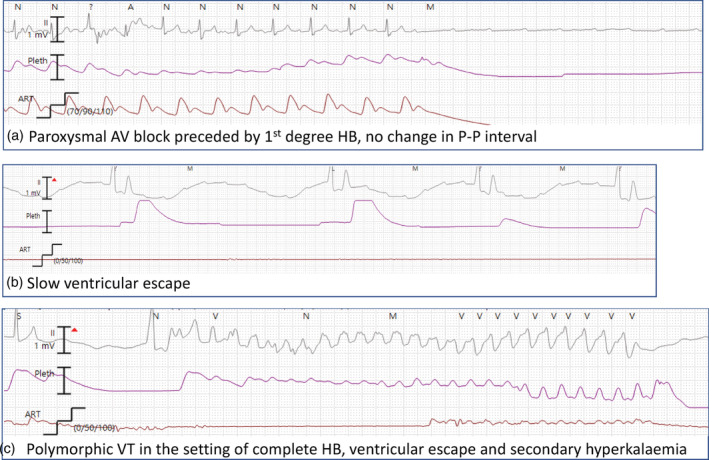
Rhythm strips taken from central monitor.

Acutely, on day 7 of admission and 1‐day post decannulation, our patient developed recurrent episodes of paroxysmal complete AV block (Fig. 2, rhythm strip A). The intermittent reduced output resulted in metabolic acidosis, secondary hyperkalaemia and a lactate rise. Prolonged ventricular pauses were recorded with an intermittent ventricular escape rhythm (Fig. 2, rhythm strip B). Resuscitation included transcutaneous synchronised ventricular pacing until dual‐chamber epicardial temporary wires could be sited via redo recent sternotomy, as is institutional preference over transvenous temporary pacing wires. A short run of ventricular tachycardia responded to medical therapy for the secondary hyperkalaemia (Fig. 2, rhythm strip C). Echocardiogram demonstrated good LV posterior wall motion, normal coronary arteries and new valvular involvement with mild mitral regurgitation. Intermittent ventricular pacing using a dual‐chamber mode with a long AV interval (250 ms) was required for 36 h until the rhythm spontaneously resolved to first‐degree AV block (AV interval < 200 ms), at which time the sternum was closed after cardiac biopsy was performed which demonstrated non‐specific inflammatory changes without evidence of myocarditis and negative SARS‐CoV‐2 PCR.

**Fig. 3:**
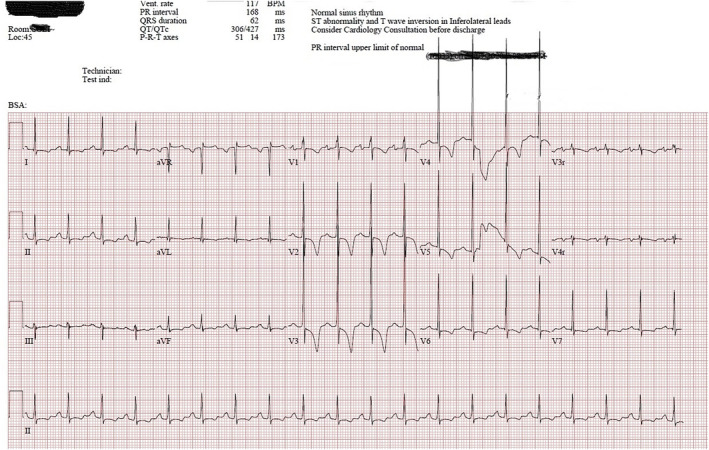
ECG prior to discharge.

By day 11 of admission, our patient was extubated and obeying commands but had profound muscle weakness and remained in first‐degree AV block. The ECG done 3 days prior to discharge showed (in addition to a borderline long PR) (Fig. 3), non‐specific ST abnormalities including lateral T inversion with minor ST depression. This prompted an echocardiogram to be performed which showed, no pericardial effusion, normal biventricular systolic function and no evidence of coronary ectasia or aneurysm formation. It was therefore judged that the findings were consistent with the non‐specific inflammatory changes seen at biopsy and further imaging (such as magnetic resonance imaging) was not required. The patient remained on aspirin at discharge and has since had outpatient follow‐up, which demonstrated a PR interval of 160 ms (between 95th and 98th centile for age),^7^ normal echocardiogram and a Holter monitor with brief episodes of Wenckebach without higher grade block. Her PR interval was normal during periods of sinus tachycardia. She has no residual deficits and has returned to school. Her most recent outpatient ECG 6 months post‐presentation showed a PR interval of 146 ms (Table 1).

**Table 1:** PR intervals

ECG				
Date	Time	HR	PR (ms)	
31‐Aug	12:21	144	152	
02‐Sep	18:24	90	NA	Junctional
03‐Sep	08:01	97	NA	Isorhythmic
08‐Sep	13:10	134	134	
09‐Sep	12:32	138	140	Pericardiac change
09‐Sep	18:16	95	184	
23‐Sep	15:29	117	168	
19‐Oct	10:32	95	152	
08‐Dec	14:04	88	150	
15‐Dec	12:03	78	140	

ECG, electrocardiogram; HR, heart rate; PR, Pwave to Rwave interval.

## Discussion

Coronavirus 2019 (COVID‐19), caused by SARS‐CoV‐2, has been described as temporally associated with a paediatric inflammatory multisystem syndrome (PIMS‐TS) also known as multisystem inflammatory syndrome associated with Coronavirus‐2019 (MIS‐C).^8^, ^9^ The WHO case definition for MIS‐C/PIMS‐TS includes fever, rash, gastrointestinal symptoms and cardiac involvement with elevated markers of inflammation and evidence of COVID‐19 infection or exposure.^4^ Over 80% of patients with PIMS‐TS present along a spectrum of cardiac diseases marked by high concentrations of troponin and brain natriuretic peptide, whereas others develop arrhythmia, left ventricular dysfunction and coronary artery dilatation or aneurysms.^1^, ^9^

While the development of conduction system abnormalities is a known complication of coronary ischemia, acute myocarditis and Kawasaki Disease with severe coronary involvement, paroxysmal complete AV block in this patient population is rare and only a recently recognised phenomenon.^2^, ^3^ Therefore, other differential diagnoses such as congenital heart disease, drugs, electrolyte imbalances and fibrosis or sclerosis of the conduction system, should be ruled out or treated where appropriate.^10^ The mechanism by which COVID‐19 can affect the myocardium and conduction system of paediatric patients is not well studied but is suspected to be secondary to inflammation and oedema. Our case adds to an evolving knowledge of MIS‐C/PIMS‐TS with cardiac features across the spectrum of pancarditis. It appears that most patient groups have a Kawasaki‐like presentation with predominant coronary artery involvement, but others may present with conduction system disease and valvar involvement, more consistent with the pancarditis seen in acute rheumatic fever.

Dionne and colleagues reported a similar case of paroxysmal AV block in a PIMS‐TS patient with no coronary artery involvement and normal PR interval at presentation.^3^ Inflammatory carditis with a risk of advanced AV block would usually demonstrate first‐degree AV block at presentation. Our patient had a PR interval of 154 m at presentation, on the 95th centile for age. Thus, it is noteworthy from both Dionne et al. and our case that all children are at risk of paroxysmal AV block in PIMS‐TS despite having a normal PR interval at presentation. Serial 12‐lead ECG's may be helpful, however may be difficult to perform in dynamic situations and in patients canulated centrally with an open sternum as in our patient. This case also highlights that haemodynamic compromise due to paroxysmal AV block can present in the convalescent stage of this novel hyperinflammatory syndrome.

It is clear from the multiple cases still being reported that there is still much to learn about PIMS‐TS and evolving presentations will continue to challenge paediatric physicians world‐wide.

## References

[1] Ramcharan T , Nolan O , Lai CY *et al*. Paediatric inflammatory multisystem syndrome: Temporally associated with SARS‐CoV‐2 (PIMS‐TS): Cardiac features, management and short‐term outcomes at a UK tertiary paediatric hospital. Pediatr. Cardiol. 2020; 41: 1391–401. 32529358 10.1007/s00246-020-02391-2 PMC7289638

[2] Domico M , McCanta AC , Hunt JL , Ashouri N , Nugent D , Kelly RB . High‐grade heart block requiring transvenous pacing associated with multisystem inflammatory syndrome in children during the COVID‐19 pandemic. HeartRhythm Case Rep. 2020; 6: 811–4. Available from: https://linkinghub.elsevier.com/retrieve/pii/S2214027120301895. 32864334 10.1016/j.hrcr.2020.08.015 PMC7446646

[3] Dionne A , Mah DY , Son MBF *et al*. Atrio‐ventricular block in children with multisystem inflammatory syndrome. Pediatrics 2020; 27: e2020009704. 10.1542/peds.2020-009704 32855347

[4] WHO . Clinical Management Clinical Management Living Guidance COVID‐19. 2021B; 2021;16–44.

[5] MacLaren G , Butt W , Best D , Donath S . Central extracorporeal membrane oxygenation for refractory pediatric septic shock. Pediatr. Crit. Care Med. 2011; 12: 133–6. 20453704 10.1097/PCC.0b013e3181e2a4a1

[6] Pan KC , Shankar S , Millar J *et al*. Role of levosimendan in weaning children requiring veno‐arterial extracorporeal membrane oxygenation after cardiac surgery. Eur. J. Cardio‐Thoracic Surg. [Internet] 2020; 59: 262–8. 10.1093/ejcts/ezaa275 32910173

[7] Davignon A . ECG Standards for Children 1980.

[8] Multisystem inflammatory Syndrome in Children and Adolescents With COVID‐19 Scientific Brief 15 May 2020 Background [Internet]; 2020. Available from: https://www.who.int/news‐room/commentaries/detail/multisystem‐inflammatory‐syndrome‐in‐children‐and‐adolescents‐with‐covid‐19

[9] Whittaker E , Bamford A , Kenny J *et al*. Clinical characteristics of 58 children with a pediatric inflammatory multisystem syndrome temporally associated with SARS‐CoV‐2. J. Am. Med. Assoc. 2020; 324: 259–69. 10.1001/jama.2020.10369 PMC7281356 32511692

[10] Brignole M , Deharo JC , Guieu R . Syncope and idiopathic (paroxysmal) AV block. Cardiol. Clin. [Internet] 2015; 33: 441–7. 10.1016/j.ccl.2015.04.012 26115830

